# Biopsy Proven Tumefactive Multiple Sclerosis with Concomitant Glioma: Case Report and Review of the Literature

**DOI:** 10.3389/fneur.2015.00150

**Published:** 2015-07-17

**Authors:** Esteban E. Golombievski, Matthew A. McCoyd, John M. Lee, Michael J. Schneck

**Affiliations:** ^1^Department of Neurology, Stritch School of Medicine, Loyola University Chicago, Maywood, IL, USA; ^2^North Shore University Health System, Evanston, IL, USA; ^3^Department of Neurosurgery, Stritch School of Medicine, Loyola University Chicago, Maywood, IL, USA

**Keywords:** tumefactive, multiple sclerosis, glial cell tumors, oligoastrocytoma, demyelination

## Abstract

We report a case of pathologically confirmed tumefactive multiple sclerosis (MS) followed shortly thereafter by the diagnosis of an oligoastrocytoma. The complexity of diagnosis and management of concomitant presence of tumefactive MS and glial cell tumors is discussed.

## Case Report

A 69-year-old man presented to our Neurosurgery Service with a 2-month history of trouble writing, poor coordination of his right hand, and word finding difficulties. Initial neurological examination was unremarkable. Medical history was remarkable for hypertension, dyslipidemia, coarctation of aorta (diagnosed at age 18 and corrected with surgery), and L4–L5 laminectomies due to significant pain. There was a strong family of cancer including a brother (deceased age 77) with glioblastoma (GBM) and a daughter (deceased age 34) with osteosarcoma (unknown location).

**Figure 1:**
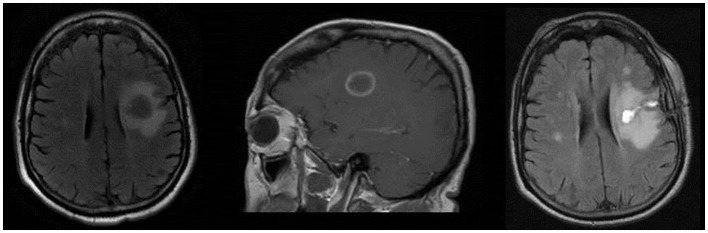
**Left image: axial T2 FLAIR showing 2 cm × 2.3 cm left frontal mass with substantial edema**. Middle image: sagittal T1 post-contrast image showing contrast enhancement in a “ring-like” pattern. Right image: axial T2 FLAIR showing residual changes consistent with partial resection of the previous ring-enhancing left frontal mass.

**Figure 2:**
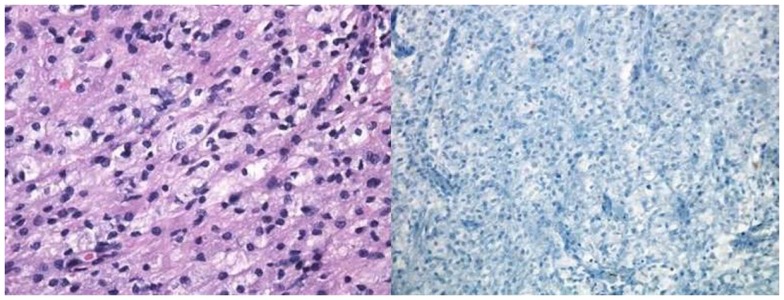
**Pathological specimen of the left frontal lobe lesion**. Left image: H&E section showing white matter with reactive gliosis (reactive astrocytes with abundant eosinophilic cytoplasm). Right image: Luxol fast blue showing absence of myelin.

Routine blood chemistries and CBC were unremarkable. MRI of the brain, with and without contrast, showed a left frontal, ring-enhancing mass measuring 2 cm × 2.3 cm with substantial edema (Figure 1). He was started on intravenous (IV) methylprednisolone 1 g daily for 5 days. He then had a craniotomy with partial resection of the mass lesion. Pathologic evaluation of the resected tissue demonstrated reactive gliosis with CD-68 macrophages most likely consistent with a demyelinating lesion (Figure 2). After surgery, the Neurology Service was consulted.

He was weaned off steroids. He experienced considerable improvement of his dexterity and ability to concentrate.

About 10 months later, an MRI of the brain was taken at follow-up, showing no evidence of local recurrence of the left frontal lesion. There were patchy hyperintensities in the right occipital and left frontal areas. At that time, he was asymptomatic, and his clinical examination was unremarkable.

**Figure 3:**
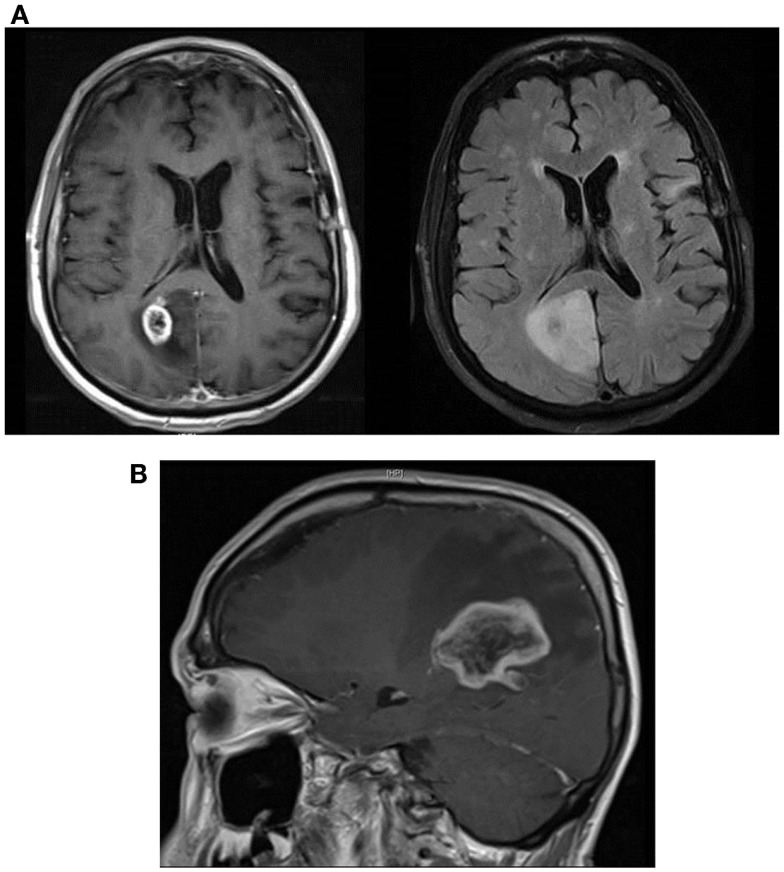
**(A)** Sagittal T1 post-contrast showing large area of right occipitoparietal lesion with a ring-like enhancing pattern (left image) and axial T2 FLAIR MRI showing substantial peri-lesional edema (right image). **(B)** Sagittal T1 post-contrast MRI showing marked increase in the size of the right parietal lobe lesion with surrounding local edema and enhancement.

Three months later, he presented with difficulty concentrating and poor short-term memory. MRI of the brain now showed increased size of the right occipital and parietal lobe lesions, with a new area of occipitoparietal ring enhancement (Figure 3A).

A 5-day course of high-dose IV methylprednisolone was started for a possible recurrent demyelinating process. Cerebrospinal fluid (CSF) analysis showed normal protein levels with no white blood cells (WBC), glucose 63 mg/dl (60%), and 0.77 IgG index (with serum IgG 622 mg/dl). Myelin basic protein (MBP) and serum neuromyelitis optica (NMO) antibodies were negative. CSF sampling for oligoclonal bands (OGB) was positive. Somatosensory evoked potentials (SSEPs) were normal and visual evoked potentials (VEPs) were uninterpretable.

He was discharged on a tapering dose of oral steroids. He was subjectively better, but became symptomatic again with generalized weakness, more prominent on the left side. The weakness slowly progressed until an initial dose of natalizumab was given. The patient then experienced abrupt worsening of symptoms. Neurological examination showed left-sided hemiparesis, left hemibody neglect, and left inferior homonymous quadrantanopia. MRI of the brain revealed an increased area of enhancement in the deep, right parietal lobe with mass effect (Figure 3B). The patient was taken to the OR for resection of the lesion. Pathology showed oligoastrocytoma; grade III on cytology. Chemotherapy with temozolamide (standard protocol) and radiation therapy (5940 cGy) in 33 fractions to the right parietal lobe lesion were initiated. The patient then developed worsening left leg weakness and further MRI of the spine showed nodular enhancing lesions of the lumbar spine consistent with leptomeningeal metastasis. At that point, bevacizumab was initiated in addition to adjustment in the dexamethasone dose. The patient unfortunately was lost to follow-up thereafter.

## Discussion

Occurrence of multiple sclerosis (MS) and brain tumors has been reported <50 times in the literature, since first described by Bosch in 1912 (1). Concurrence of MS and cerebral glial cell tumors is uncommon, but has been described (2, 3). Whereas some authors believe that tumors develop from neoplastic transformation of reactive glial cells in the involved area of demyelination, others believe that this is purely coincidental (2–7). Tumors and plaques are not contiguous in all reported cases and multicentricity, similar to MS plaques, is reported in about 30% of these tumors (8, 9). In most cases, MS is the initial pathology with subsequent development of cerebral glioma, most frequently of the astrocytic subtype type (6).

Tumefactive demyelinating lesions (TDLs) are defined as large (>2 cm) demyelinating lesions occurring either as solitary lesions or as a few separated lesions with little vasogenic edema, and incomplete or open ring-enhancing lesions (10, 11). Like brain neoplasms, TDLs enhance and exhibit mass effect, often making it difficult to differentiate TDL from neoplasm.

MRI is the most sensitive technique for depicting demyelinating disease. A study by Kim and colleagues (12) showed that comparing the corresponding areas of CT hypo-attenuation with the respective MR enhancing regions is specific for distinguishing TDL from primary glioma, or CNS lymphoma. Combined modalities of MR and non-contrast CT are significantly more accurate than MRI imaging alone for differentiating these entities (12). Magnetic resonance spectroscopy (MRS) can differentiate between astrocytic tumors and demyelinating lesions by showing (not only in MS but also in low-grade gliomas) increased myoinositol peaks (indicative of reactive gliosis) and decreases in *N*-acetylaspartate peaks (indicative of neuronal cell loss). Tumors also show decreases in *N*-acetylaspartate but have typical increased choline peaks (indicating rapid mitotic activity) and increased lactate peaks (indicative of intratumoral glycolysis) (13). When lesions manifest with tumefaction mimicking brain gliomas, or CNS lymphoma, the diagnosis will still be made by histopathological studies showing foamy macrophages, reactive gliosis with lack of myelin in MS and variable degrees of high-cell density, mitosis, nuclear atypia, microvascular proliferation, and necrosis with gliomas (14).

Management of high-grade gliomas often requires cytoreductive therapy followed by adjuvant therapy with radiotherapy (XRT) and/or chemotherapy (15). Additional morbidity may occur when giving XRT to a patient with glioma who has a concomitant demyelinating disorder (16). Patients with MS and glioma have survival-times identical to those observed in patients not suffering from MS. Moreover, the coexistence of MS and brain tumor does not influence the clinical evolution of either of these pathologies, and despite extensive area of demyelination, the course of TDLs is rather benign with unpredictable progression to MS as compared to the course of brain gliomas (11, 17).

## Author Note

This case submission met all of the requirements for publication under the rules of the Loyola University Stritch School of Medicine Institutional Review Board.

## Conflict of Interest Statement

The authors declare that the research was conducted in the absence of any commercial or financial relationships that could be construed as a potential conflict of interest.
